# Supplementary material for: Content-rich biological network constructed by mining PubMed abstracts
Source: BMC Bioinformatics. 2004 Oct 8;5:147. doi: 10.1186/1471-2105-5-147 (PMC528731; doi:10.1186/1471-2105-5-147)
Supplement: Additional File 2 — The original results of the above study (non-essential files are deleted to keep the file size under the limit set by BMC bioinformatics). [file 1471-2105-5-147-S2.bz2 › chilibotAdditionalFile2/dip30/689ID9294424E18009/html/SECA_SECY.html]

 


 **SECA** and **SECY** 
  
Found 153 abstracts in PubMed, retrieved 30.  
 

 What does Google say? 
 PDF only 
| .edu only 

---

**Interactive relationship** (e.g. stimulation, inhibition, etc)

**Neutral relationship**- It is suggested that the C terminal  **SecY**  residues are required for  **SecA**  dependent translocation initiation.  Ref: 11914356 J Bacteriol, 2002
- The fifth and the sixth cytoplasmic regions C5 and C6 of  **SecY**  are important for the  **SecA**  driven preprotein translocation reaction.  Ref: 12351621 J Biol Chem, 2002
- CONCLUSIONS The Walker motif A regions of  **SecA**  are important for the  **SecA**   **SecY**  interaction that leads to the  **SecA**  conformational changes required for insertion into the SecYEG channel.  Ref: 11168585 Genes Cells, 2000
- In concert with SecB,  **SecA**  targets the precursors to  **SecY**  and energizes their translocation by its ATPase activity.  Ref: 11051763 Prog Nucleic Acid Res Mol Biol, 2001
- **SecY**  F286Y supports only a low  **SecA**  binding affinity, preprotein translocation and  **SecA**  translocation ATPase activity.  Ref: 11755523 FEBS Lett, 2002
- Genetic analysis of  **SecA**   **SecY**  interaction required for spore development in Bacillus subtilis.  Ref: 10713435 FEMS Microbiol Lett, 2000
- We now show that a  **secY**  mutation, which compromises a functional  **SecY**   **SecA**  interaction, does not impair the SRP mediated integration of polytopic inner membrane proteins.  Ref: 10931878 J Cell Biol, 2000
- Characterization of a mutant form of  **SecA**  that alleviates a  **SecY**  defect at low temperature and shows a synthetic defect with  **SecY**  alteration at high temperature.  Ref: 10833277 J Biochem Tokyo , 2000
- The data also suggest that the conformation of the central region of  **SecA**  is important for the regulation of ATP hydrolysis and for the productive interaction of  **SecA**  with  **SecY** .  Ref: 10833277 J Biochem Tokyo , 2000
- Thus, this mutant provides an excellent system for genetic analysis of the  **SecY**   **SecA**  interaction.  Ref: 11168585 Genes Cells, 2000
- The former class of mutations, presumably with specific effects on the  **SecY**   **SecA**  interaction, clustered in two regions close to the Walker motif A sequences of the two ATP binding domains.  Ref: 11168585 Genes Cells, 2000
- We introduced random mutations into a segment of Escherichia coli  **SecY**  within its cytoplasmic domain 5, which was shown previously to be important for the  **SecA**  dependent translocation activity.  Ref: 11309495 Proc Natl Acad Sci U S A, 2001
- In Escherichia coli, three different types of proteins engage the  **SecY**  translocon of the inner bacterial membrane for translocation or insertion 1 polytopic membrane proteins which prior to their insertion into the membrane are targeted to the translocon using the bacterial signal recognition particle SRP and its receptor, 2 secretory proteins which are targeted to and translocated across the  **SecY**  translocon in a  **SecA**  and SecB dependent reaction and 3 membrane proteins with large periplasmic domains, requiring SRP for targeting and  **SecA**  for the translocation of the periplasmic moiety.  Ref: 12682042 J Biol Chem< MedlineTA>J Biol Chem, 2001
- A mutation in  **secY**  that causes enhanced  **SecA**  insertion and impaired late functions in protein translocation.  Ref: 10852867 J Bacteriol, 2000

**Non-interactive relationship** (e.g. studied together, co-existance, homology, etc.)

- Here, we use a cross linking approach to show that hydrophilic portions of a co translationally targeted inner membrane protein FtsQ are close to  **SecA**  and  **SecY** , suggesting that insertion takes place at the  **SecA**  Y interface.  Ref: 10675323 EMBO J, 2000
- One set is more similar to  **SecA**  and  **SecY**  of Escherichia coli.  Ref: 12010500 Mol Microbiol, 2002
- Crosslinking experiments showed that the hydrophilic region P1 of nascent membrane inserted Lep 100 mer was in the vicinity of  **SecA**  and  **SecY** .  Ref: 10913619 FEBS Lett, 2000
- Genes encoding  **SecA**  and  **SecY**  proteins, essential components of the Sec protein translocation system, were cloned from onion yellows phytoplasma, an unculturable plant pathogenic bacterium.  Ref: 11551069 Mol Plant Microbe Interact, 2001
- The mass and structure of this SecYEG as well as the subunit stoichiometry of  **SecA**  and  **SecY**  in a soluble translocase precursor complex reveal that translocase consists of the  **SecA**  homodimer and four SecYEG complexes.  Ref: 10698927 EMBO J, 2000
- These results indicate that SRP dependent integration and  **SecA**  SecB mediated translocation do not only represent two independent protein delivery systems, but also remain mechanistically distinct processes even at the level of the membrane where they engage different domains of  **SecY**  and different components of the translocon.  Ref: 10931878 J Cell Biol, 2000
- Most bacterial species have a single copy of the genes encoding  **SecA**  and  **SecY** , which are essential for viability.  Ref: 12010500 Mol Microbiol, 2002
- Nascent Lep inserts into the Escherichia coli inner membrane in the vicinity of YidC,  **SecY**  and  **SecA** .  Ref: 10913619 FEBS Lett, 2000
- However, Streptococcus gordonii strain M99 encodes  **SecA**  and  **SecY**  homologues that are not required for viability or for the translocation of most exported proteins.  Ref: 12010500 Mol Microbiol, 2002
- Pulse chase experiments showed that significant amount of wild type RBP was processed in a  **secY**  mutant in the presence of azide  **SecA**  inhibitor.  Ref: 12810088 Biochem Biophys Res Commun, 2003
- For both substrates, the mRNA levels of  **secA** , secD, secE, secF, and  **secY**  genes were almost constant during the early and log phases, but showed a marked decrease at the beginning of the stationary phase followed by a full recovery of mRNA level in the late stationary phase.  Ref: 10696484 Can J Microbiol< MedlineTA>Can J Microbiol, 2003
- At least two other Gram positive species, Streptococcus pneumoniae and Staphylococcus aureus, encode two sets of  **SecA**  and  **SecY**  homologues.  Ref: 12010500 Mol Microbiol, 2002
- Besides  **SecA**  and SecB, Escherichia coli cells possess a signal recognition particle SRP to target exported proteins to the  **SecY**  translocon.  Ref: 10619852 EMBO J, 2000
- Once targeted to  **SecY** , ribosome associated nascent chains of the hybrid protein remain untranslocated unless  **SecA**  is present.  Ref: 11101515 EMBO J, 2000
- Previously, we have identified a cold sensitive  **secY**  mutation secY205, affecting the most carboxy terminal cytosolic domain, that did not allow an ATP dependent insertion of a  **SecA**  preprotein complex.  Ref: 11168585 Genes Cells, 2000
